# Determination of Frequency of the Second Mesiobuccal Canal in the Permanent Maxillary First Molar Teeth with Magnification Loupes (× 3.5)

**Published:** 2014-09

**Authors:** Muhammad Hasan, Farhan Raza Khan

**Affiliations:** Dental Section, Department of Surgery, The Aga Khan University Hospital, Karachi, Pakistan

**Keywords:** Mesiobuccal canal, permanent maxillary first molar, magnification

## Abstract

**INTRODUCTION:**

The mesiobuccal root of the maxillary first molar has generated more research and clinical investigation than any root. An inability to detect and treat a second mesiobuccal (MB2) canal is a reason for endodontic failure in maxillary first molars. Modifications in the endodontic access and detection techniques, along with advancements in illumination and magnification technology, have aided in the location and treatment with the second mesiobuccal canal of maxillary first molars.

**OBJECTIVE:**

To determine the frequency of the second mesiobuccal canal in the permanent maxillary first molars with magnification loupes (× 3.5).

**MATERIALS AND METHODS:**

A total of 53 teeth were assessed using a moderate magnification for second mesiobuccal canal in mesiobuccal root of first permanent maxillary molars *in vivo.* Detection of this canal in maxillary first molars was done through a clinical access cavity preparation under magnification loupes (× 3.5). Data was analyzed using SPSS 15.0. Frequency distribution of variables was determined and the level of significance was kept at 0.05.

**RESULTS:**

We were able to detect second mesiobuccal canal in 27 out of 53 (50.9%) of the permanent maxillary first molars that were studied. It was found that the males tend to have a higher proportion of second mesiobuccal canals (up to 31%) as compared to the females in whom the second mesiobuccal canals could be identified only 19% of the time. Whilst, there was no association found between age, gender and chamber obliteration with the presence of second mesiobuccal canal.

**CONCLUSIONS:**

In conclusion, within its limitations, this study suggested that the use of magnification loupes enhanced both the detection (50.9%) and negotiation (86.8%) of the second mesiobuccal canals in the permanent maxillary first molars beyond what could be achieved with naked eye.

## INTRODUCTION

The mesiobuccal root of the maxillary first molar has generated more research and clinical investigation than any root (1). The reason being that the mesiobuccal root of the maxillary molar comprises of an additional root canal known as the second mesiobuccal canal (2). This canal is one of the most frequently missed root canal in maxillary molar teeth. This is because in the maxillary first molars, the second mesiobuccal canal departs chamber at a sharp mesial inclination and then bend again distally, which makes its detection and negotiation challenging (3). Furthermore, an inability to detect and treat a second mesiobuccal (MB2) canal may be a reason for endodontic failure in maxillary first molars (4). Nonetheless, endodontically retreated teeth were found to contain more undetected MB2 canals than first-time treated teeth, suggesting that failure to treat existing MB2 canals leads to a poorer prognosis (5).

Modifications in endodontic access and detection techniques, along with advancements in illumination and magnification technology, have aided in the location and treatment of the second mesiobuccal canal of maxillary first molars (6). By using an improved access technique alone Weller and colleagues, in his retrospective clinical study, recorded a second mesiobuccal canal in 39% of his sample of maxillary first molars and 21.4% in the maxillary second molars (6). The improved technique included the creation of a rhomboidal shape to access preparation outline and a thorough probing of the groove between the mesial and palatal canals with a sharp endodontic explorer (7). Ultrasonics is used by few endodontists for the purpose of MB2 canal search, and use of bur and explorer is preferred by the majority. A retrospective clinical study performed by Hartwell and Bellizzi recorded four canals in 18% and 9% of first and second maxillary molars, respectively. This percentage of second mesiobuccal canals located proved to be a significant increase from the earlier reported results (7).

Using magnification during endodontic treatment has particular advantages. It increases the confidence level of the operator by improving control during troughing and searching in the deep chambers of maxillary molars thereby reducing significant risk of perforations. Moreover, magnification further enhances the ability of the operator to effectively search for the second mesiobuccal canal and as a result leads to higher number of such canals being located and treated (8). Eventually, this may lead to higher quality endodontic treatment. Likewise, controlled troughing under magnification is important and advantageous. This is because in the maxillary molars, the trough helps eliminate the first angled portion of the canal, allowing insertion of instruments beyond the bend.

There is scarcity of data on the frequency of second mesiobuccal canals in maxillary first molars in our country; therefore, we have planned this study to explore the frequency of MB2 with magnification loupes in our setting.

## MATERIALS AND METHODS

This was a cross descriptive sectional study conducted in the dental clinics of Aga Khan University Hospital, Karachi. Pulp chambers were inspected in a total of 53 patients during treatment. Non-probability purposive sampling was used in this study. The inclusion criteria were permanent maxillary first molar teeth of male and female patients between 7 years to 70 years of age. Patients were recruited after obtaining a detailed history, clinical examination and correlating the radiographic findings. Diagnosis for root canal treatment was confirmed by obtaining history of spontaneous pain (irreversible pulpitis), acute or chronic apical periodontitis, acute or chronic periapical abscess and failed previous root canal treatment (presenting with similar signs and symptoms). On examination, if there was pulpal involvement along with periapical infection around the roots; the patient was advised root canal treatment. When the patient agreed for the treatment, a written consent was obtained and treatment was initiated. The exclusion criteria consisted of patients who refused to provide informed consent, patients presenting with teeth having incompletely formed apices, severely calcified canals, resorptive defects, complex root anatomy, severe periodontal or concomitant endo-perio disease as well as patients with restricted mouth opening (<three finger breadths).

The sample size was calculated using a statistical calculator “Sample Size Determination in Health Studies, WHO”. The reported incidence of the MB2 canal with × 3.5 magnification is 63% (outside our region) (7). We anticipated the same proportion of MB2 canal in our population (detected with the help of magnification), i.e. incidence on magnification (P) = 63% and confidence level = 95%, the sample size requirement turned out to be of 53 patients.

### Data collection procedure

A total of 53 teeth were assessed using a moderate magnification [EyeMag Pro F, Carl Ziess Meditec Ag, Germany] for second mesiobuccal canal in mesiobuccal root of first permanent maxillary molars *in vivo*. Detection of the second mesiobuccal canal in the mesiobuccal root of maxillary first molars was done through a clinical access cavity preparation using magnification [× 3.5]. The treatment began with the administration of 2% Xylestesin with 1:80,000 dilution of epinephrine. A rubber dam was applied and access cavity was prepared in a rhomboidal shape following the law of concentricity (10) by using a medium round bur in high speed handpiece. Upon completing access cavity, the pulpal tissue was extirpated and the pulp chamber floor was evaluated for canal orifices.

**Figure 1:**
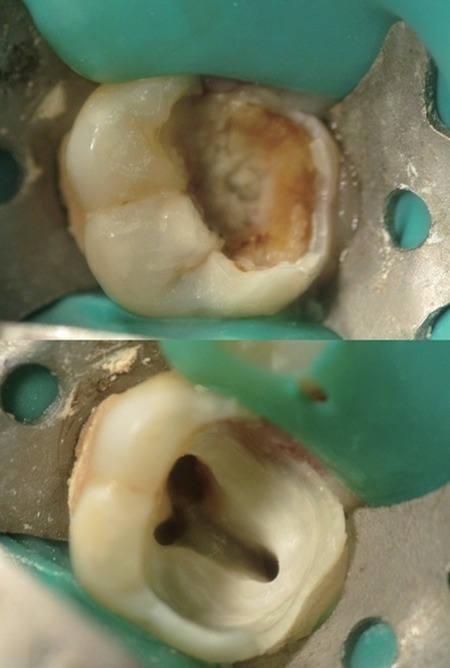
An actual case of maxillary first molar. a, A carious maxillary first molar; b, Access opening showing mesiobuccal canals.

**Figure 2:**
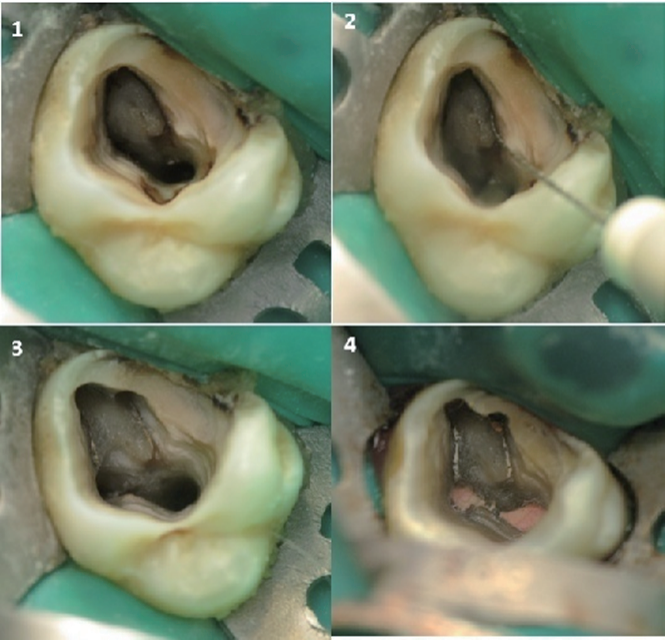
Another example of a case demonstrating second mesiobuccal canal. 1, Access opening of maxillary first molar; 2, MB 2 located; 3, All canals prepared and ready for obturation; 4, The post-obturation view.

The primary mesiobuccal, distobuccal and palatal orifices were identified and scouted using small hand files. At this stage, a working length radiograph was obtained. The identified canals were coronally flared and the access was further refined so that the orifices lie at the peripheries of the chamber and the triangular dentin lying on top of each orifice is also eliminated. If the second mesiobuccal canal could not be readily located, gentle troughing with a small round carbide bur in low speed handpiece was performed on the mesial sub-pulpal groove. This developmental groove forms a line that connects the palatal and mesiobuccal canals and the orifice of mesiobuccal canal is usually present on this groove or 2 mm mesial to it. After at least 2 mm of chamber floor troughing, if the second mesiobuccal could still not be located, then no further effort was made. This was done to prevent any inadvertent perforation. Examples of actual clinical cases can be seen in Figure 1 and Figure 2. Standard root canal treatment was then performed which involved thorough shaping, cleaning and obturating of the root canal system followed by a definitive restoration.

### Ethical Considerations

Patients were informed about their participation in the research study which involves a standard root canal treatment with no extra charge to them. Their identities were kept strictly confidential. Patients were further informed regarding the risk of perforation during troughing for additional root canal. After having a mutual agreement, the informed consent forms were signed by the patients and the treatments were undertaken. The Ethical Review Committee of the hospital approved the protocol before recruitment of patients to the study.

### Data Analysis

Data was analyzed using SPSS 19.0. Frequency distribution of variables was determined. For qualitative variables i.e. gender, principal diagnosis, canal configuration, number of second mesiobuccal canals, bar and pie charts were made. For quantitative variables i.e. age, canal length, mean and standard deviation were computed. Chi square test was applied to determine any association between the presences of second mesiobuccal canal with other variables. Level of significance was kept at 0.05.

## RESULTS

The total number of patients in this study was 53 with the mean age of 34.9 (± 11.89) years. As shown in Table 1, both genders were almost equally represented in the sample (male 26 and females 27). Left molars (n=31) were more frequently encountered than right side (n=22). In the clinical presentation of cases, teeth with chronic caries (n=48) had the maximum proportion comprising of 90%, followed by 4% cases of acute caries (n=2), 2% cases of heavily restored teeth (n=2), 2% cases of perio-endo lesion (n=2), and similarly 2% cases were referred for dental extraction (n=2) out of all the 53 cases. In 72% of the cases, the pulpal spaces were found patent on opening (n=38), while in the remaining 28% (n=15) of the cases, pulpal spaces were found calcified. The main outcome variable; frequency of second mesiobuccal canal found was about 50.9% (n=27) out of a total of 53 cases evaluated. While in the remaining 49% of the cases (n=26), this second mesiobuccal canal could not be located. The percentage of teeth found with the presence of the second mesiobuccal canal was 50.9 % (27 teeth out of total 53 teeth). This was highly statistically significant with a *p*-value of <0.001. Table 2 shows the association of all the evaluated independent variables with the presence of second mesiobuccal canal. Chi-square test was applied at 0.05 level of significance. In 32% of the males (17 out of 26), the presence of the second mesiobuccal canal was confirmed while in remaining 17% (9 out of 26) this canal could not be located. In contrast, 19% of the females (10 out of 27) were found to have this second mesiobuccal canal while in 32% of the females (17 out of 27) this second mesiobuccal canal could not be located. With regards to the presence of the second mesiobuccal canal, the difference found in males and females was statistically significant with a *p*-value of 0.039. There was no statistically significant difference found with regards to the location of the tooth in the maxillary arch (*p*-value 0.90), clinical presentation (*p*-value 0.80), patency of the pulpal space (*p*-value 0.317), diagnosis (*p*-value 0.915), and with the presence of the orifice (*p*-value 1.0). The presence of orifice explains that when located, the MB2 canal was negotiated to working length in 86.8% of the cases i.e. 33 orifices located but 27 canals could further be negotiated.

**Table 1:** Distribution of Independent Variables (n=53)

Variables	Categories	Counts & (%)

Gender	Female	27 (51)
	Male	26 (49)
Location	Left maxillary	31 (59)
	Right maxillary	22 (42)
Clinical presentation	Chronic caries	48 (90)
	Acute caries	2 (4)
	Heavily restored tooth	1 (2)
	Perio-Endo lesion	1 (2)
	Referred for extraction	1 (2)
	Suspected vertical root fracture	0 (0)
Pulpal space	Patent	38 (72)
	Calcified	15 (28)
Diagnosis	Symptomatic irreversible pulpitis with acute apical periodontitis	20 (37.7)
	Necrotic pulp	18 (35.8)
	Non-surgical retreatment	6 (11.3)
	Asymptomatic irreversible pulpitis with chronic periodontitis	4 (7.5)
	Elective Endodontics	4 (7.5)
	Trauma	0 (0)
Orifice opening present but non- negotiable	Yes	7 (13.2)
Total number of teeth	53	

**Table 2:** Association of Variables With Second Mesiobuccal Canal

s. no	VARIABLES		MB2 Counts (%)		*p*-value
			Absent	Present	

1	Gender	Male	9 (17)	17 (32)	**0.039 **
		Female	17 (32)	10 (19)	
2	Location	Right	11 (21)	11 (21)	0.90
		Left	15 (28)	16 (30)	
3	Clinical Presentation	Acute caries	1 (2)	1 (2)	0.80
		Chronic caries	23 (43)	25 (47)	
		Heavily Restored	1 (2)	0	
		Endo-Perio Lesion	1 (2)	0	
		Referred For Extraction	1 (2)	1 (2)	
4	Pulp space	Normal	17 (32)	21 (40)	0.317
		Calcified	9 (17)	6 (11)	
5	Diagnosis	Symptomatic Irreversible Pulpitis With Acute Apical Periodontitis	11 (21)	9 (17)	0.915
		Asymptomatic Irreversible Pulpitis With Periodontitis	2 (4)	2 (4)	
		Necrotic Pulp	9 (17)	10 (19)	
		Elective Endodontics	1 (2)	3 (6)	
		Nonsurgical Retreatment	3 (6)	3 (6)	
6	Opening present but not negotiable	yes	6 (11)	1 (2)	1.00

Chi-square test was applied at 0.05 level of significance.

**Table 3:** Comparison of various studies with our center’s study

Reference	Number of teeth	Type of study	1 canal %	2 canal %

Pomeranz, Fishelberg (1974) (24)	71	Clinical RCT^a^	72.0% (51)	28.0% (20)
Slowey, RR (1974) (40)	103	Clinical radiographic examination	49.6% (51)	50.4% (52)
Sempira, HN and Hartwell, GR (2000) (12)	130	Clinical RCT^a^ using SOM^b^ or loupes	66.9% (87)	33.1% (43)
Buhrley, LJ *et al* (2002) (6)	208	Clinical RCT^a^ using SOM^b^ or loupes	29.9% (62)	71.1% (148)
Wolcott, J *et al* (2002) (3)	1193	Clinical examination of RCT^a^ treated and retreated teeth	39.0% (465)	61.0% (728)
Present study (2012)	53	Clinical RCT^a^ and using loupes	49.1% (26)	50.9% (27)

a Root Canal Treatment.

b Surgical Operating Microscope.

## DISCUSSION

There are studies in the present pool of research that associate failed root canal treatment of permanent maxillary molars to the missed accessory canals (most frequently the second mesiobuccal canal) but the evidence is still somewhat deficient, however, still most clinicians accept this empirical notion that failure to locate and treat the MB2 canal may lead to a poor prognosis (9-15). In a study done by Wolcott *et al.* (5), the criteria for endodontic failure, and thus indication for retreatment when restorable, was based on the criteria originally proposed by Bender *et al*. (16) and updated in the Quality Assurance Guidelines published by the American Association of Endodontists (17-20). It was for the first time acknowledged by Weine (21) that the failure of endodontic treatment of maxillary molars is likely because of the inability to locate and fill the second mesiobuccal canal.

In this study, magnification was not compared with the naked vision because currently magnification is considered vital when performing endodontic treatment (22). Therefore, magnification loupes (× 3.5) were utilized to search for the second mesiobuccal canal in permanent maxillary first molars. Buhrley *et al* (23) recently reported that the incidence of finding this canal in three distinct groups of practitioners was 17%, 63% and 71%. The first group used no magnification, the second group wore dental loupes, and the third used an operating microscope. In their study, investigators determined that dental loupes and the DOM were equally effective for locating the MB2 canal in maxillary molars (23). One Jordanian *in vitro* study concluded that, the MB2 canal orifice could be detected through a clinical access cavity with careful use of a bur (troughing) as high as 56.7% of the time. The use of magnification increased the rate to 63.9%, although the difference was not statistically significant. The prevalence of MB2 canals was high (77.3%). The effectiveness of detecting those canals was 73.3% without loupes and 82.7% with loupes (24). Previous* in vivo* studies have investigated the incidence of the MB2 canal in maxillary molars; however, none of these studies indicated whether or not any form of magnification was used. The majority of *in vivo *researches have shown an incidence of the MB2 canal ranging from 18% to 36% (9, 25). Three studies showed an incidence of 64.6%, 77.2%, and 52% (26-29). Readers are referred to table 3 for a better comparison of our findings to a few* in vivo* studies.

Stropko considered a second mesiobuccal canal present if the author was simply able to instrument the canal to a depth of 3 to 4 mm after troughing (30). Furthermore, he stated that approximately 9% of the MB2 canals could not be fully instrumented and were thought to be rudimentary canals that did not exist in the apical one-half of the root (30). Kulild and Peters (10) found the same problem of non-negotiability of the second mesiobuccal canals to occur 45.8% of the time, whereas Neaverth *et al.* demonstrated an even higher rate of 61.8% which is highly significant (19). In our study, if the orifice was located then attempts were given to negotiate it using #08 and #10 files. If the files entered the orifice more than 5 mm then the second mesiobuccal canal was taken as present. If less than 4 mm length was achieved then the orifice was labeled as an opening present but non-negotiable. However, our selection criteria were more stringent and we did not count such incomplete canals as this may lead to an overestimation of the frequency. Whenever a MB2 orifice was located in our study, we were able to negotiate it to working length in 86.8% of the cases i.e. 33 orifices located out of which 27 canals could further be negotiated to a length of 4 mm or more than or equivalent to 4 mm.

The second mesiobuccal canals in maxillary permanent molars come under the category of hidden canals. In this study, additional techniques that were employed by the operator also play an important role in successfully locating these hidden canals. Nevertheless, there could be many difficulties encountered when conducting such *in vivo* studies for determination of root canal frequencies. One is the unpredictability of the teeth as they have many variations that are difficult to evaluate preoperatively. Some teeth have more aberrant internal anatomy and pose more difficulty in treatment than others. Another concern is that some clinicians may be less inclined to trough and search a hidden canal search hidden canals as opposed to others. It is possible that, in some instances, the most important factor in locating the MB2 canal is not the magnification but the persistence of a motivated operator who holds a philosophical belief.

With the use of magnification, the frequency of the MB2 canal in our study was almost twice that of the majority of previous international *in vivo* studies. It is more in agreement with the findings of Green (25). Furthermore; it has been shown that the use of magnification leads to a MB2 detection rate as much as three times higher than when not utilizing magnification (25, 31, 32). Although, not related to the detection of the second mesiobuccal canal, pulp vitality testing was performed on each tooth. This was done to reflect the type of cases that are frequently encountered at our center. On vitality testing, up to 38% cases had symptomatic irreversible pulpitis with acute apical periodontitis. The second most common diagnosis was pulpal necrosis which was 36%. Around 11% of the cases presented with post-treatment disease requiring non-surgical endodontic retreatment.

## CONCLUSIONS

Based on the study results, it seems that magnification of the operating field provided by the loupes is an important factor in successfully locating the MB2 canal. Males are more likely to exhibit these canals than females. However, this may prove to be an insignificant finding in a study with a higher sample size. One particular limitation in this study was the absence of a second examiner who could reassess each case in which the primary operator had failed to identify a second mesiobuccal canal. Therefore, authors believe that having a second evaluator may result in reporting of a higher frequency of second mesiobuccal canals than this study.

## References

[1] Cleghorn BM, Christie WH, Dong C (2006). Root and root canal morphology of the human permanent maxillary first molar: a literature review. J. Endod.

[2] Stropko JJ (1999). Canal morphology of maxillary molars: clinical observations of canal configurations. J. Endod.

[3] Gorduysus MO, Gorduysus M, Friedman S (2001). Operating microscope improves negotiation of second mesiobuccal canals in maxillary molars. J. Endod.

[4] Henry BM (1993). The fourth canal: its incidence in maxillary first molars. J. Can. Dent. Assoc.

[5] Wolcott J, Ishley D, Kennedy W, Johnson S (2005). A 5 yr clinical investigation of second mesiobuccal canals in endodontically treated and retreated maxillary molars. J. Endod.

[6] Weller RN, Hartwell GR (1989). The impact of improved access and searching techniques of detection of the mesiolingual canal in maxillary molars. J. Endod.

[7] Hartwell GR, Bellizzi R (1982). Clinical investigation of *in vivo* Endodontically treated mandibular and maxillary molars. J. Endod.

[8] Yoshioka T, Kikuchi I, Fukumoto Y, Kobayashi C (2005). Detection of the second mesiobuccal canal in mesiobuccal roots of maxillary molar teeth ex vivo. Int. Endod. J.

[9] Pomeranz HH, Fishelberg G (1974). The second mesiobuccal canal of the maxillary molars. J. Am. Dent. Assoc.

[10] Kulild JC, Peters DD (1990). Incidence and configuration of canal systems in the mesiobuccal root of maxillary first and second molars. J. Endod.

[11] Fogel HM, Peikoff MD, Christie WH (1994). Canal configuration in the mesiobuccal root of the maxillary first molar: a clinical study. J. Endod.

[12] Weller RN, Niemczyk SP, Kim S (1994). Incidence and position of the canal isthmus. Part 1. Mesiobuccal root of the maxillary first molar. J. Endod.

[13] Acosta SA, Trugeda SA (1978). Anatomy of the pulp chamber floor of the permanent maxillary first molar. J. Endod.

[14] Caliskan MK, Pehlivan Y, Sepetcioglu F, Turkun M (1995). Root canal morphology of human permanent teeth in a Turkish population. J. Endod.

[15] Henry BM (1993). The fourth canal: its incidence in maxillary first molars. J. Can. Dent. Assoc.

[16] Bender IB, Seltzer S, Soltanoff W (1966). Endodontic success-a reappraisal of criteria. Part II. Oral Surg. Oral Med. Oral Pathol.

[17] Vertucci FJ (1984). Root canal anatomy of the human permanent teeth. Oral Surg. Oral Med. Oral Pathol.

[18] Seidberg BH, Altman M, Guttuso J, Suson M (1973). Frequency of two mesiobuccal root canals in maxillary permanent first molars. J. Am. Dent. Assoc.

[19] Neaverth EJ, Kotler LM, Kaltenbach RF (1987). Clinical investigation (*in vivo*) of endodontically treated maxillary first molars. J. Endod.

[20] (1998). Appropriateness of Care and Quality Assurance Guidelines.

[21] Weine FS, Healey HJ, Gerstein H, Evanson L (1969). Canal configuration in the mesiobuccal root of the maxillary first molar and its endodontic significance. Oral Surg. Oral Med. Oral Pathol.

[22] Dankner E, Friedman S, Stabholz A (1990). Bilateral C shape configuration in maxillary first molars. J. Endod.

[23] Buhrley LJ, Barrows MJ, BeGole EA, Wenckus CS (2002). Effect of magnification on locating the MB2 canal in maxillary molars. J. Endod.

[24] Smadi L, Khraisat A (2007). Detection of a second mesiobuccal canal in the mesiobuccal roots of maxillary first molar teeth. Oral Surg. Oral Med. Oral Pathol. Oral Radiol. Endod.

[25] Green D (1973). Double canals in single roots. Oral Surg. Oral Med. Oral Pathol.

[26] Seidberg BH, Altman M, Guttuso J, Suson M (1973). Frequency of two mesiobuccal root canals in maxillary permanent first molars. J. Am. Dent. Assoc.

[27] Neaverth EJ, Kotler LM, Kaltenbach RF (1987). Clinical investigation (*in vivo*) of endodontically treated maxillary first molars. J. Endod.

[28] Nosonowitz DM, Brenner MR (1973). The major canals of the mesiobuccal root of the maxillary 1st and 2nd molars. NY J. Dent.

[29] VandeVoorde HE, Odendahl D, Davis J (1975). Molar 4th canals: frequentcause of endodontic failure?. Ill Dent. J.

[30] Stropko JJ (1999). Canal morphology of maxillary molars: clinical observations of canal configurations. J. Endod.

[31] Alavi AM, Opasanon A, Ng YL, Gulabivala K (2002). Root and canal morphology of Thai maxillary molars. Int. Endod. J.

[32] Brown P, Herbranson E (2004). Dental anatomy & 3D tooth atlas version 2.0.

